# Prevalence and multilocus genotyping of potentially zoonotic *Giardia duodenalis* in pigs in Shanghai, China

**DOI:** 10.1017/S0031182019000349

**Published:** 2019-05-14

**Authors:** Hua Liu, Ning Xu, Jianhai Yin, Zhongying Yuan, Yujuan Shen, Jianping Cao

**Affiliations:** National Institute of Parasitic Diseases, Chinese Center for Disease Control and Prevention; Chinese Center for Tropical Diseases Research; WHO Collaborating Centre for Tropical Diseases; National Center for International Research on Tropical Diseases, Ministry of Science and Technology; Key Laboratory of Parasite and Vector Biology, MOH; Shanghai 200025, China

**Keywords:** Assemblage, *bg*, *gdh*, *Giardia duodenalis*, *tpi*

## Abstract

*Giardia duodenalis* is a common intestinal parasite in humans and other mammals, and it causes major public and veterinary health problems worldwide. China is a major pig-raising country, and studies on *Giardia* in pigs have important public health significance. The present study was conducted to investigate the prevalence of *Giardia* and assess its genetic characterization. A total of 93 samples were collected from two farms in Shanghai. The presence of *Giardia* was determined using PCR and sequence analysis of glutamate dehydrogenase, beta-giardin and triose phosphate isomerase genes. The average prevalence of *G. duodenalis* infection was 26.88% (25/93) in the pigs, with 28.13% (18/64) in farm 1 *vs* 24.14% (7/29) in farm 2. All the PCR-positive products were successfully sequenced, and assemblage E was more prevalent. Zoonotic assemblages A and B and canine-specific assemblage C were identified in farm 1, whereas, only assemblage E was detected in farm 2. Interestingly, two pig isolates showed 100% homology with human-derived isolates from Australia and China at the *bg* and *tpi* loci respectively. Pigs infected with *Giardia* infect humans by polluting the environment; whether pigs are a potential environmental source of the human pathogen in China requires more epidemiological data.

## Introduction

*Giardia duodenalis* (syn. *Giardia lamblia* and *Giardia intestinalis*) is an important intestinal protozoan that infects a wide range of mammalian species, for example, humans, wildlife, livestock and companion animals such as dogs and cats (Xu *et al*., 2016; Li *et al*., 2017*b*). Generally, infection with *Giardia* results in self-limited illness with weight loss and malabsorption, and asymptomatic giardiasis is common in developing countries (Hellard *et al*., 2000; Thompson, 2000). In 2004, giardiasis was classified as a neglected tropical disease by WHO because of its adverse effects on the growth and cognition development of children (Savioli *et al*., 2006). Giardiasis has a significant public health impact and affects veterinary health. The genus *Giardia* is divided into eight assemblages/genotypes (A to H) on the basis of genetic analysis. The zoonotic assemblages A and B can infect humans and many mammalian species, such as wild animals, nonhuman primates, domestic animals and companion animals. Other assemblages are more host-specific. Assemblages C and D infect domestic and wild canines, assemblage E infects domestic ruminants and pigs and assemblage F infects cats. Assemblage G is mostly found in rodents, and assemblage H, in seals (Cacciò *et al*., 2018). However, some of these assemblages have also been identified in humans, such as assemblage F in children living under poor environmental conditions in Slovakia, assemblage E in people living in Australia and assemblage C in diarrhoea patients in Shanghai, China (Liu *et al*., 2014*b*; Zahedi *et al*., 2017; Pipiková *et al*., 2018). In fact, transmission of *Giardia* from humans to animals or vice versa has been detected in areas where humans have close contact with animals such as lambs (Traub *et al*., 2004; Lebbad *et al*., 2010). A previous study has also shown the possibility of sexual transmission of *Giardia* in endemic areas (Escobedo *et al*., 2018).

In humans, the number of giardiasis cases has been estimated to be about 28.5 million, with an average infection rate of 2.52%. The annual incidence of Giardia infection accounts for more than 10% of the total number of cases worldwide (Li *et al*., 2017*b*). In China, large-scale investigations of *G. duodenalis* in humans showed infection rates of 6.04% (81/1332) in Huainan, Anhui Province, and 9.46% in Shanghai (Fu *et al*., 2004; Wang *et al*., 2013). The infection rate of Giardia is higher in HIV/AIDS patients, with the highest rate of up to 16.2% in Guangzhou, China (Pand *et al*., 2015). In China, assemblages A and B are the main genotypes of Giardia in humans (Li *et al*., 2017*a*). Recently, assemblage C was identified as the predominant species in diarrhoea patients in Shanghai, China (Liu *et al*., 2014*b*), and assemblage E was identified to have a high infection rate (6.8%, 6/88) in humans in Queensland, Australia (Zahedi *et al*., 2017).

In animals, the prevalence of giardiasis varies greatly in different countries because of the animal species, sample methods, environment and development status. The infection rate has been reported to be as high as 52% in cattle in the United States and 66.4% in pigs in Canada (Hoar *et al*., 2009; Farzan *et al*., 2011). Recently, studies on *Giardia* in animals have been performed in at least 27 provinces and autonomous regions in China, with a prevalence rate of 0.51–50% in non-human primates, 1.04–22.6% in cattle, 0–27.78% in sheep and goats, 3.71–31.51% in dogs and cats, 1.7–11.1% in wild boar and domestic pigs, 1.9–8.38% in rabbits and 6.03–37.50% in rodents (Fan *et al*., 2017; Li *et al*., 2017*a*, 2017*b*; Wang *et al*., 2018; Zhang *et al*., 2018). Molecular methods have been used in several studies, and assemblages A and B have been isolated from animals. Assemblages A and E were found in a dog and cattle, respectively; assemblage B, in rabbits; assemblages C, D and F, in companion dogs and assemblage G, in racehorses (Li *et al*., 2017*a*, 2017*b*). The molecular studies of *Giardia* in animals in China mainly concentrated on cattle, sheep, cats and dogs. There is limited information on the prevalence and genetic characterization of *G. duodenalis* in pigs. Pigs are one of the main sources of meat products in China. Swine manure may cause environmental contamination through the water or other ways (Thurston-Enriquez *et al*., 2005), and a large number of *Giardia* spores in animal slurry can also enter streams and rivers from pasture run-off. In 2011, researchers investigated pollution by *Cryptosporidium* and *Giardia* in the water source of the Huangpu River and showed through genotyping that pigs are one of the sources of pollution (Feng *et al*., 2011). Therefore, it is essential to study *Giardia* in pigs in the area around Huangpu River.

Although several genes, such as small-subunit (SSU) rRNA, glutamate dehydrogenase (gdh), triose phosphate isomerase (tpi) and beta-giardin (bg), are widely used to identify Giardia with PCR, a single gene may not accurately identify Giardia or fully describe its genetic characterization. Multilocus genotyping (MLG) based on more than three genes is being used to provide more genetic information and contribute to understanding possible zoonotic transmission linkages (Cacciò *et al*., 2008; Scorza *et al*., 2012). The aim of the present study was to assess the prevalence of *G. duodenalis* in pigs from two farms in Shanghai, which is the largest economic centre of the country, by amplification of gdh, bg and tpi and sequencing and investigate the possible zoonotic potential of *G. duodenalis* at a genetic level.

## Materials and methods

### Sample collection

In the two farms, permission to conduct the study was obtained from the managers. In 2014, a total of 93 faecal samples were collected from the farms. The samples were collected from freshly dropped faeces by using a sterile disposable latex glove, placed in clean plastic bags, transported on ice to the laboratory and stored at 4 °C until DNA extraction.

### DNA extraction

The faecal samples were washed three times using sterile water, and genomic DNA was extracted using the QIAamp DNA Stool Mini Kit (Qiagen, Valencia, USA), according to the manufacturer's protocol. The DNA was eluted in 200 *µ*L of AE elution buffer and stored at−30 °C until use.

### Molecular methods

All the samples were analysed for the three loci. A 530 bp fragment of *gdh*, 530 bp fragment of *tpi* and 380 bp fragment of *bg* were amplified using nested PCR (Cacciò *et al*., 2002; Sulaiman *et al*., 2003; Scorza *et al*., 2012). For all three genes, primary PCR was performed with 12.5 *µ*L of 2 × PCR master mix (Promega, Italy), 1 *µ*L of each primer (10 *µ*m), and 1 *µ*L of DNA in a total reaction volume of 25 *µ*L. For the nested PCR, 1 *µ*L of the first PCR product was used as the template. The PCR cycling conditions were the same for *gdh* and *tpi*: initial hot start at 95 °C for 5 min, followed by 35 cycles (94 °C for 50 s, 57 °C for 45 s and 72 °C for 1 min) and a final extension step at 72 °C for 10 min. The secondary PCR cycling conditions were identical to the primary PCR cycling conditions. For *bg*, the cycling conditions were the same, except the annealing temperature was 60 °C. A *Giardia*-positive DNA specimen and distilled water were used as the positive and negative controls, and the PCR products were analysed using 2% agarose gel electrophoresis and ethidium bromide staining.

### DNA sequencing and data analysis

For accurate analysis, all the genes were amplified at least three times and all PCR-positive products were sequenced in both directions using an ABI 3730 DNA Analyzer (Applied Biosystems, Foster City, USA), secondary primers and a Big Dye Terminator v3.1 Cycle Sequencing kit (Applied Biosystems). ContigExpress was used to evaluate the wave peak and assemble the sequences. The nucleotide sequences were aligned and edited using BLAST, BioEdit (version 7.0.9), GenBank and ClustalX 1.83 (ftp://ftp-igbmc.u-strasbg.fr/pub/ClustalX/).

## Results

### Prevalence and PCR amplification of *G. duodenalis*

**Fig. 1.**
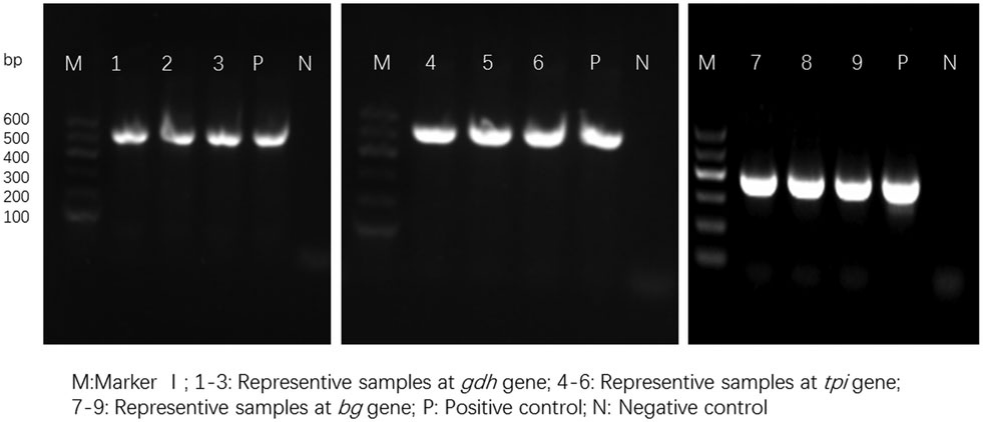
PCR results of representative samples for the *gdh*, *tpi* and *bg* genes.

For the 93 samples, nested PCR amplification of *gdh*, *tpi* and *bg* was performed. On the basis of at least one gene, the average prevalence of *G. duodenalis* infection was 26.88% (25/93) in the pigs (Table 1). In farm 1, *gdh*, *bg* and *tpi* were detected in 11 (17.19%), 11 (17.19%) and 5 (7.81%) samples, respectively. Among the samples, all three loci were successfully amplified in two isolates, whereas *gdh* and *bg* were amplified in five isolates. On the basis of one locus, *gdh*, *bg* and *tpi* were successfully amplified in four, four and three isolates. In farm 2, *gdh*, *bg* and *tpi* were detected in five (17.24%), three (10.34%) and two (6.90%) samples. Among the samples, the three loci were successfully amplified in only one isolate, whereas *gdh* and *bg* were amplified in one isolate. In total, the prevalence of *G. duodenalis* was 28.13% (18/64) in farm 1 *vs* 24.14% (7/29) in farm 2, and *gdh* showed a higher amplification rate (17.20%, 16/93) than *bg* (15.05%, 14/93) and *tpi* (7.53%, 7/93). PCR results of representative samples for the *gdh*, *tpi* and *bg* genes are shown in Fig. 1.

**Table 1.** Infection rate and assemblage distribution of *G. duodenalis* in pigs

Farm	Prevalence^a^	gdh		tpi		Bg		Total assemblages^b^
		No. of positive (%)	Assemblage (n)	No. of positive (%)	Assemblage (n)	No. of positive (%)	Assemblage (n)	
Farm 1	28.13% (18/64)	11 (17.19)	A (2), E (9)	5 (7.81)	C (2), E (3)	11 (17.19)	A (1), B (1), E(9)	A (2), B (1), C (2), E(13)
Farm 2	24.14% (7/29)	5 (17.24)	E (5)	2 (6.90)	E (2)	3 (10.34)	E (3)	E (7)
Total	26.88% (25/93)	16 (17.20)	A (2), E (14)	7 (7.53)	C (2), E (5)	14 (15.05)	A (1), B (1), E(12)	A (2), B (1), C (2), E(20)

a Prevalence based on one locus.

b Total assemblages indicating that if one isolate had the same assemblage at different loci, it was considered as one assemblage.

### Percentage and distribution of *G. duodenalis* assemblages

In this study, assemblages A, B, C and E were found in the pigs. Using the gdh locus, 16 specimens were identified to be assemblage A and 14, assemblage E, whereas, using the tpi locus, two specimens were identified to be assemblage C and five, assemblage E. The expected fragment of bg was successfully amplified in 14 specimens, and assemblage A (1), assemblage B (1) and assemblage E (12) were identified. In general, assemblage E was predominant in the pigs in the investigated areas. This result is consistent with those obtained in China and other countries.

In addition, the different farms yielded different genotypes. In farm 1, the genotypes were diverse, and assemblages A, B, C and E were identified. However, in farm 2, only assemblage E was detected.

### Homology analyses of *G. duodenalis* assemblages

Sequence comparison with *G. duodenalis* sequences available in the GenBank database revealed that the isolates listed in Table 2 showed 100% homology with the sequences reported previously. One isolate was confirmed to be assemblage A by amplification of *gdh* (KJ668144) and *bg* (KJ668152), which have been reported in the grey seal (GU176079) from the United States and ferret (AB469365) from Japan, respectively. The other assemblage A isolate was identical to a cattle-derived isolate (KF843930) from China. The assemblage B isolate (KJ668151) showed 100% homology with human-derived isolates from Australia (HQ179586). Two assemblage C isolates were identical to a dog-derived isolate from the United States (AY228641) and a human-derived isolate from China (KF271451). Six (KJ668142) and four (KJ668138) assemblage E isolates have been found in pig- (AY178741) and cattle-derived (AY178740) isolates from Australia. On the basis of the *tpi* locus, two isolates were identical to cattle-derived isolates from Japan and the United States. On the basis of the *bg* loci, eight isolates have been described in pigs from the Czech Republic (AY072729) and one in sheep from the United States (DQ116624).

**Table 2.** Homology analyses of pig-derived isolates of *G. duodenalis* assemblages

Assemblage	Accession no. (This study)	Loci amplified	Country	Host	Accession no.	Ref
A	KJ668143	*gdh*	China	Cattle	KF843930	Unpublished
	KJ668144	*gdh*	USA	Grey seal	GU176079	Lasek-Nesselquist *et al*. (2010)
	KJ668152	*bg*	Japan	Ferret	AB469365	Unpublished
B	KJ668151	*bg*	Australia	Human	HQ179586	Wielinga *et al*. (2011)
C	KJ668133	*tpi*	USA	Dog	AY228641	Minetti *et al*. (2014)
	KJ668131	*tpi*	China	Human	KF271451	Liu *et al*. (2014*b*)
E	KJ668142	*gdh*	Australia	Pig	AY178741	Unpublished
	KJ668138	*gdh*	Australia	Cattle	AY178740	Unpublished
	KJ668137	*gdh*	China	Sheep Cattle	KC960647 KF843925	Liu *et al*. (2014*a*). Unpublished
	KJ668130	*tpi*	Japan	Cattle	AB569406	Suzuki *et al*. (2011).
	KJ668132	*tpi*	USA	Cattle	EF654692	Feng *et al*. (2008)
	KJ668149	*bg*	Czech Republic	Pig	AY072729	Cacciò *et al*. (2002)
	KJ668146	*bg*	USA	Sheep	DQ116624	Di Giovanni *et al*. (2006)

### Genetic diversity of assemblage E

In addition to the above-mentioned sequences, other assemblage E isolates were analysed, and a multiple alignment was performed (Table 3). In this study, intra-genotypic diversity of *G. duodenalis* assemblage E was observed. On the basis of the *bg* loci, three subtypes were noted using AY072729 as the reference sequence (Table 3). On the basis of the *tpi* loci, the obtained isolates could also be divided into three subtypes, with five- to six-base variations at seven nucleotide sites. The subtyping analysis revealed that three isolates have not been described on the basis of the *gdh* loci, and a detailed description of the single nucleotide polymorphisms is provided in Table 3.

**Table 3.** *G. duodenalis* assemblage E subtypes on the basis of the *gdh, bg* and *tpi* loci

Accession no.	*bg*										
	Position	86	101	236	242	266					
AY072729		A	T	G	T	C					
KJ668150		G	C	G	C	T					
KJ668147		G	T	G	C	C					
KJ668148		G	C	A	T	T					
	*tpi*										
	Position	21	94	106	347	348	456	474			
JF792419		T	G	G	A	T	A	A			
KJ668134		T	A	A	G	G	G	G			
KJ668135		C	A	G	G	G	G	G			
KJ668136		T	A	G	G	G	G	G			
	*gdh*										
	Position	65	83	89	125	194	266	334	439	461	464
AY178741		T	C	C	G	A	G	C	A	G	G
KJ668145		C	T	C	G	G	T	C	G	G	G
KJ668141		T	C	C	G	G	G	T	A	A	G
KJ668138		T	C	T	A	G	T	C	G	G	G

## Discussion

Pigs are complicated hosts of many diseases caused by bacteria, viruses and parasites. Globally, *Giardia* assemblages A, B, C, D, E and F have been identified in pigs, with assemblage E being the predominant species (Wang *et al*., 2018). In the United Kingdom and Australia, pigs have been implicated as sources of *G. duodenalis*, and zoonotic genotypes occur frequently. However, only a few studies have investigated the infection and molecular epidemiology of *Giardia* in pigs in China (Wang *et al*., 2017, 2018; Shi *et al*., 2018), which is a major pig-raising country. Recently, African swine fever was reported in pigs in different cities in China, causing wide public concern (Ge *et al*., 2018). Therefore, considering that pigs are the main economic animals in China and the importance of zoonotic *Giardia*, we performed multilocus genotyping of *G. duodenalis* in pigs. To the best of our knowledge, this is the first report of the occurrence and genetic characterization of giardiasis in pigs in Shanghai, China. *Giardia* spp. were identified in 26.88% (25/93) of the pigs by using nested PCR, with 28.13% (18/64) in farm 1 *vs* 24.14% (7/29) in farm 2. In this study, the infection rate was higher than that detected in pigs in Henan (8%), Sichuan (3.1%), Shanxi (1.7%) and Yunnan (1.55%) and lower than that reported in Canada (50.8%), Western Australia (31.1%) and the United Kingdom (57.1%) (Armson *et al*., 2009; Farzan *et al*., 2011; Minetti *et al*., 2014; Wang *et al*., 2017, 2018; Shi *et al*., 2018). The results showed that the amplification rate of *gdh* was higher (16, 17.20%) than that of *bg* (14, 15.05%) and *tpi* (6, 6.45%). In fact, infection rates are complicated and related to many factors, such as the selected locus, detection methods, different seasons and farms and the structure of the specimens (Geurden *et al*., 2008). In addition, a different management system, involving differences in animal stocking density, water supply or hygiene regimes, could increase the potential risk of infection by intestinal parasites like *Giardia*.

Globally, few studies on the genotyping of *Giardia* have been conducted, with the infection rate being 0–66.4%. The genotypes are mainly assemblages A, B, C, E and F with assemblage E being predominant, except for a study in Canada, in which assemblage B was the main genotype (Farzan *et al*., 2011; Wang *et al*., 2017). In this study, the obtained sequences were all aligned with reference sequences; the specimens were determined to be assemblages A, B, C or E, with assemblage E being more prevalent. This is consistent with the results of previous studies conducted in other countries (Langkjaer *et al*., 2007; Armson *et al*., 2009), but different from those of a study performed in Ontario, Canada (Farzan *et al*., 2011). No assemblage swapping was found in the specimens (different assemblages at different loci in the same isolate). Interestingly, different assemblages were found at the two farms. Assemblages A, B, E and canine-specific assemblage C were identified at farm 1, and only assemblage E was found at farm 2. This may be because the two farms are in different parts of Shanghai, with farm 1 in the middle of the city and farm 2 in southwestern Shanghai. In addition, the difference in the number of samples from the farms could have influenced the results, as only 29 specimens were collected from farm 2.

All the assemblage A isolates have been described previously in different hosts. The sequence analysis showed that two isolates typed as assemblages B and C in the study were identical to the human-derived isolates on the basis of *bg* and *tpi*, respectively (Wielinga *et al*., 2011; Liu *et al*., 2014*b*). Similar to our study, assemblage C was found in a pig from the United Kingdom (Minetti *et al*., 2014). Unexpectedly, the assemblage C isolate (KJ668131) has been reported in diarrhoea patients in the investigated area (Liu *et al*., 2014*b*). Thus, the occurrence of assemblages A, B and C isolates is a potential zoonotic risk for humans. In our study, the molecular epidemiological data showed that assemblage E was the most common in the pigs in the investigated areas, and similar results have been reported in many countries (Maddox-Hyttel *et al*., 2006; Langkjaer *et al*., 2007; Armson *et al*., 2009; Sprong *et al*., 2009).

Sequence analysis of the *bg* locus of *G. duodenalis* revealed three subtypes in 12 assemblage E isolates, with two to four nucleotide variations; AY072729 was used as the reference sequence. Intra-genotype variations were also found on the basis of the *tpi* locus, and three novel isolates had only one or two nucleotide variations within seven sites. However, using JF792419 as the reference sequence, the single nucleotide polymorphisms increased to five or six sites, suggesting that the novel subtypes may represent endemic genetic characterizations in the investigated areas. On the basis of the *gdh* locus, 11 of 14 isolates have been reported in different animals, and three novel subtypes have been reported for the first time in pigs in Shanghai.

Currently, molecular analysis is being widely used to identify *G. duodenalis* in pigs (Table 4). In Australia, although assemblage E was the most common *Giardia* genotype, zoonotic assemblage A and feline-specific assemblage F were identified in pigs, with two mixed infections (A + E) (Armson *et al*., 2009). Similarly, assemblage F and a canine-specific assemblage C isolate were found in the United Kingdom (Minetti *et al*., 2014). In Denmark, assemblage E and zoonotic assemblage A have been identified (Langkjaer *et al*., 2007; Petersen *et al*., 2015). In Canada and Poland, assemblage E and zoonotic assemblage B have been identified (Farzan *et al*., 2011; Stojecki *et al*., 2015). In the study, two pig-derived isolates were also typed as assemblage C on the basis of *tpi*. The occurrence revealed that the host-adopted assemblages were no longer confined to specific hosts. Likewise, the canine-specific assemblage D was also found in pigs from Demark and Europe, whereas assemblage E was more prevalent. In China, *Giardia* assemblages A, B, C, E and F have been reported in Shanxi, Yunnan, Henan and Sichuan Provinces (Li *et al*., 2017*a*, 2017*b*; Wang *et al*., 2017, 2018; Shi *et al*., 2018). In fact, contaminated water, food and fomites are considered to be the sources of infection for *G. duodenalis* (Feng and Xiao L, 2011). To our knowledge, the pig farms involved in our study were not industrialized pig farms and faeces may pollute the environment through water or other routes during the treatment process; similar findings have been reported by Hutchison *et al*. (2004). In addition, children or adults in close contact with farm animals are at increased risk of *Giardia* infection (Hoque *et al*., 2002, 2003).

**Table 4.** Assemblage distribution of *G. duodenalis* isolates from pigs worldwide

Country	Loci amplified	No. of samples with assemblage				Reference
		A	B	E	Others	
Australia	18SrRNA	17		35	1(F), 2(A+E)	Armson *et al*. (2009)
Brazil	*gdh*			2	1(D)	Fava *et al.* (2013)
	*tpi*			1		
Demark	Unspecified	10		52	1(D)	Langkjaer *et al*. (2007) Maddox-Hyttel *et al*. (2006)
Europe	Unspecified	29	1	109	1(D)	Sprong *et al*. (2009)
Italy	SSUrRNA+*gdh*+*tpi*+*bg*	1				Lalle *et al*. (2005)
Canada (Ontario)	SSU rRNA, bg		58	5		Farzan *et al*. (2011)
UK	SSUrRNA				1(C), 1(F)	Minetti *et al*. (2014)
China	*tpi*	3	0	9	3(C)	Wang *et al*. (2018)
China	*MLGs*	9	0	36		Wang *et al*. (2017)
China	TPI		2			Shi *et al*. (2018)
China	*bg*	2	0	9		Feng *et al*. (2011)
China	*gdh*	2		14		This study
	*tpi*			5	2(C)	
	*bg*	1	1	12		

In 2005, three Egyptians were identified to be infected with *G. duodenalis* assemblage E, which was the first report in humans (Foronda *et al*., 2008). In view of the aforementioned infection factors, the pigs infected with *G. duodenalis* in the two farms may also be considered as a potential source of infectious cysts that affect humans. Because our study was a cross-sectional survey, and the farms were selected on the basis of the willingness of the owners, our data may not reflect the true population prevalence of *G. duodenalis* in farm animals in Shanghai. However, MLG based on three loci was used to detect the specimens, so our results demonstrate that *G. duodenalis* is a common intestinal parasite of pigs in the investigated areas. Previous studies have illustrated the difficulties of confirming the assemblage of an isolate by using MLG with different loci; however, the detection method provides clues for understanding assemblage exchange and potential zoonotic transmission (Lebbad *et al*., 2010; Beck *et al*., 2012; Scorza *et al*., 2012). Recently, Cacciò *et al*. (2008) proposed an MLG model for easily defining *G. duodenalis* assemblage A isolates, and it can be used for appropriate nomenclature of sub-assemblages (subtypes) based on *gdh*, *tpi* and *bg*. Moreover, MLG has been used to identify *G. duodenalis* assemblages and sub-assemblages in humans, proving it can provide more information on the genetic diversity and transmission dynamics of *G. duodenalis* (Alyousefi *et al*., 2013; Huey *et al*., 2013).

In conclusion, to the best of our knowledge, this is the first report on pig giardiasis in Shanghai, China, and *Giardia* assemblage E was prevalent in the pigs in the investigated area. The occurrence of zoonotic assemblages A and B was also detected. In addition, the canine-specific assemblage C was found in two pigs. The finding that pig-derived assemblages B and C have 100% homology with human-derived *G. duodenalis* isolates at the *bg* and *tpi* loci implies the possibility of zoonotic transmission in the investigated areas. A better understanding of the distribution of *Giardia* in animals will help to establish more targeted measures for its prevention and control. Further studies with a larger number of samples and farms, and evaluation of the farmers in contact with pigs, are needed to investigate *G. duodenalis* infection and transmission dynamics and assess the zoonotic risk for humans.
